# Performance Improvement of TiO_2_ Ultraviolet Photodetectors by Using Atomic Layer Deposited Al_2_O_3_ Passivation Layer

**DOI:** 10.3390/mi15111402

**Published:** 2024-11-20

**Authors:** Yao-Tsung Yang, Shih-Chin Lin, Ching-Chiun Wang, Ying-Rong Ho, Jian-Zhi Chen, Jung-Jie Huang

**Affiliations:** 1 Design and Materials for Medical Equipment and Devices, Da-Yeh University, Changhua 515006, Taiwan; union9900@gmail.com; 2 Mechanical and Mechatronics System Research Labs, Industrial Technology Research Institute, Hsinchu 310401, Taiwan; shihchin@itri.org.tw (S.-C.L.); juin0306@itri.org.tw (C.-C.W.); 3 Department of Electrical Engineering, Da-Yeh University, Changhua 515006, Taiwan; yingrong0702@gmail.com; 4 Graduate Institute of Photonics, National Changhua University of Education, Changhua 50007, Taiwan; benson900320@yahoo.com.tw

**Keywords:** TiO_2_ nanorod (NR), Al_2_O_3_, ultraviolet (UV) photodetector, atomic layer deposition (ALD)

## Abstract

This study employed atomic layer deposition (ALD) to fabricate an Al_2_O_3_ passivation layer to optimize the performance of ultraviolet (UV) photodetectors with a TiO_2_-nanorod-(NR)-containing active layer and a solid–liquid heterojunction (SLHJ). To reduce the processing time and enhance light absorption, a hydrothermal method was used to grow a relatively thick TiO_2_-NR-containng working electrode. Subsequently, a 5-nm-thick Al_2_O_3_ passivation layer was deposited on the TiO_2_ NRs through ALD, which has excellent step coverage, to reduce the surface defects in the TiO_2_ NRs and improve the carrier transport efficiency. X-ray photoelectron spectroscopy revealed that the aforementioned layer reduced the defects in the TiO_2_ NRs. Moreover, high-resolution transmission electron microscopy indicated that following the annealing treatment, Al, Ti, and O atoms diffused across the interface between the Al_2_O_3_ passivation layer and TiO_2_ NRs, resulting in the binding of these atoms to form Al–Ti–O bonds. This process effectively filled the oxygen vacancies in TiO_2_. Examination of the photodetector device revealed that the photocurrent-to-dark current ratio exhibited a difference of four orders of magnitude (10^−4^ to 10^−8^ A), with the switch-on and switch-off times being 0.46 and 3.84 s, respectively. These results indicate that the Al_2_O_3_ passivation layer deposited through ALD can enhance the photodetection performance of SLHJ UV photodetectors with a TiO_2_ active layer.

## 1. Introduction

Ultraviolet (UV) photodetectors are widely used in various fields, including industry, healthcare, and defense. The materials and structure of the active layer in UV photodetectors considerably influence the sensitivity and stability of these devices. Most currently available UV photodetectors are based on inorganic compounds, and multiple studies have developed various nanostructures to enhance the performance of UV photodetectors. Commonly used two-dimensional (2D) thin-film materials in UV photodetectors include AlN, GaN, Ga_2_O_3_, ZnO, and TiO_2_ [1,2,3,4,5,6]. In addition, scholars have explored the use of one-dimensional (1D) nanostructures, such as nanowires, nanorods (NRs), and nanotubes, to enhance photodetector performance [7,8,9]. Given the superior light-harvesting capability and shorter electron transport pathways of 1D structures compared with those of 2D structures, the present study selected TiO_2_ NRs as the active layer to fabricate UV photodetectors.

To achieve effective light absorption, the active layer of the UV photodetector should comprise a relatively thick film. Common vacuum deposition techniques, such as sputtering [10,11], atomic layer deposition [12,13], and chemical vapor deposition [14,15], provide stable film quality with easily controllable parameters; however, the film deposition rate in these methods is typically low. Alternatively, numerous studies have utilized nonvacuum hydrothermal methods [16,17] to synthesize TiO_2_ NRs; these methods are cost-effective and enable rapid film growth. Nevertheless, relevant studies have reported that TiO_2_ NRs synthesized through hydrothermal methods often exhibit oxygen vacancy defects [18], which can degrade the performance of UV photodetectors. Research has indicated that TiO_2_ NRs can be coated with a thin passivation layer to effectively reduce their oxygen vacancies. For instance, one study used the liquid-phase deposition method to deposit Al_2_O_3_ passivation layers on TiO_2_ NRs in UV photodetectors [18]. This method enabled excellent step coverage to be achieved on 1D TiO_2_ NRs and effectively passivated their defects, thereby reducing the switch-off time from 26.5 to 16.5 s during device switching. Moreover, electrochemical deposition has been employed to fabricate TiO_2_ NRs/Au/PTTh UV photodetectors [19], which exhibit adequate step coverage and a high detectivity of 1.6 × 10^10^ Jones. The performance comparison of the UV photodetector studies is shown in Table 1. However, because hydrothermal methods are nonvacuum processes, they result in the generation of a higher number of defects in the passivation layer than vacuum processes. In addition, the sputtering method has been used to fabricate AlN passivation layers [20] for UV photodetectors with TiO_2_ NRs. This method offers the advantages of few defects and easy film thickness control, and it reduces the switch-off time of the photodetector to 4.32 s. However, the sputtering process exhibits poor step coverage when the TiO_2_ NRs have high aspect ratios. Thus, a more suitable method is required for coating passivation layers on TiO_2_ NRs.

**Figure 1:**
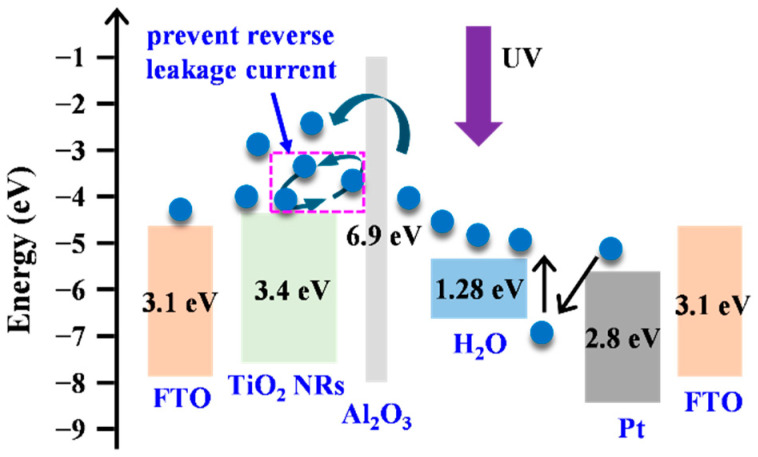
The energy-level diagram of Al_2_O_3_/TiO_2_ NR UV photodetectors.

A passivation layer should have a low thickness, few defects, the ability to repair film surface defects, and excellent step coverage. Therefore, the present study employed ALD to deposit a high-quality Al_2_O_3_ passivation layer on the TiO_2_-NR-containing active layer of UV photodetectors with a solid–liquid heterojunction (SLHJ). Figure 1 illustrates the energy-level diagram of the SLHJ UV photodetectors in this work. Al_2_O_3_ can be used to provide surface passivation to reduce the number of oxygen vacancy defects on the TiO_2_ NRs surface and can prevent reverse leakage current in SLHJ UV photodetectors, thereby enhancing the performance of photodetectors. Because a sufficient supply of oxygen atoms is maintained in the ALD process, this process can decrease the number of oxygen vacancy defects that are inherently present in the TiO_2_ NRs, thereby enhancing the quality of the working electrode of the UV photodetector. This study explored the atomic diffusion between an Al_2_O_3_ passivation layer and a TiO_2_-NR-containing working electrode after thermal treatment. The performance of the SLHJ UV photodetectors with Al_2_O_3_/TiO_2_ NR structures was also examined.

## 2. Experiment

**Figure 2:**
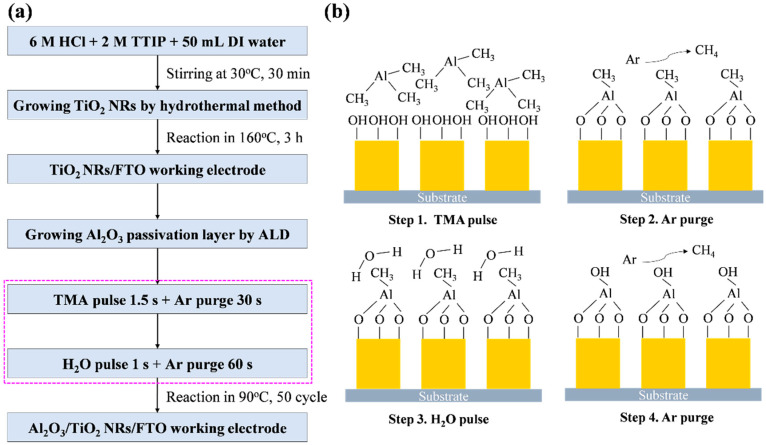
(**a**) Experimental process used to fabricate a working electrode composed of Al_2_O_3_/TiO_2_ NRs and (**b**) growth mechanism of Al_2_O_3_ passivation layer.

The process used in this study to fabricate the Al_2_O_3_/TiO_2_ NR working electrode is illustrated in Figure 2a. Initially, TiO_2_ NRs were synthesized using a hydrothermal method. A solution of 6 M HCl and 2 M titanium isopropoxide (TTIP) was prepared. This solution was then combined with deionized water in a Teflon-lined autoclave, in which a fluorine-doped tin oxide (FTO) glass substrate (area: 2 cm × 2 cm, resistivity: 15.5 Ω/m^2^) was placed. The autoclave was heated in a circulating oven at 160 °C for 3 h to obtain a working electrode. Next, the 1-μm TiO_2_ NRs/FTO was removed, rinsed with deionized water, and dried using nitrogen gas. Subsequently, ALD was employed to deposit an Al_2_O_3_ passivation layer on the TiO_2_ NRs, with trimethylaluminum (TMA) and ultrapure water used as the precursor and oxidizing agent, respectively. During the deposition process, the reaction temperature was set to 90 °C, the process pressure was maintained at 6 × 10^−2^ Torr, and Ar gas at a flow rate of 100 sccm was used as the purge gas. Each ALD cycle involved the following steps in sequence: TMA injection for 1.5 s, Ar gas purging for 30 s, H_2_O injection for 1 s, and Ar purging for 60 s. The ALD growth mechanism is shown in Figure 2b. A total of 50 ALD cycles were performed to grow a 5-nm-thick Al_2_O_3_ passivation layer on the TiO_2_ NRs, which resulted in the formation of an Al_2_O_3_/TiO_2_ NRs/FTO working electrode. Next, the Al_2_O_3_ passivation layer was subjected to annealing treatments at 300 °C, 400 °C, 500 °C, and 600 °C to enhance its properties, with the heating rate set at 30 °C/min and the temperature hold time set at 1 h. Finally, a Pt/FTO counter electrode was prepared, and a 60-μm-thick spacer (SX1170-60, Solaronix, Aubonne, Switzerland) was placed between the Al_2_O_3_/TiO_2_ NRs/FTO and Pt/FTO electrodes. The spacer was melted by applying pressure on a 100 °C hot plate to bind the working electrode (photoelectrode) and the counter electrode substrate. Deionized water was then injected to create a UV photodetector with SLHJ.

The experimental characterizations conducted in this study were as follows. First, the surface morphologies of the Al_2_O_3_ and TiO_2_ NR films were analyzed through field-emission scanning electron microscopy (JEOL JSM-7000F, Tokyo, Japan) under an accelerating voltage of 15 kV. Second, the microstructures of the TiO_2_ NRs coated with Al_2_O_3_ were examined through high-resolution transmission electron microscopy (HR-TEM, model: JEM-2100 F, Japan). Third, the chemical compositions and bonding of the TiO_2_ active layer and Al_2_O_3_ passivation layer were investigated through X-ray photoelectron spectroscopy (XPS; PHI 5000 VersaProbe, Japan) with Al Kα radiation (photon energy of 1486.6 eV). The energy resolution of the adopted XPS instrument was 0.5 eV full width at half maximum. XPS measurements were conducted at a base pressure of 7.4 × 10^−7^ Pa in an analyzer chamber. A 2-kV argon ion beam with a current density of 100 A/cm^2^ was used to acquire the depth profiles, and the binding energy of each element was calibrated to that of the C1s (284.5 eV) peak. Finally, the current (I)–voltage (V) characteristics of the created photodetector under UV illumination (illumination intensity and wavelength of 15 W and 365 nm, respectively) and dark conditions were explored using the HP 4145B Semiconductor Parameter Analyzer.

## 3. Results and Discussion

**Figure 3:**
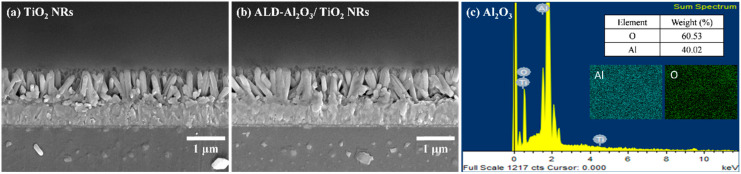
Field-emission scanning electron microscopy images of the surface morphologies of (**a**) TiO_2_ NRs, (**b**) Al_2_O_3_/TiO_2_ NRs, and (**c**) EDS analysis of ALD-Al_2_O_3_.

**Figure 4:**
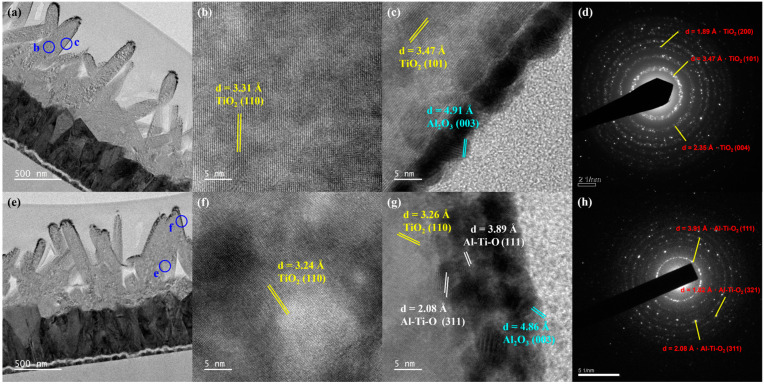
Cross-sectional high-resolution transmission electron microscopy images of (**a**) Al_2_O_3_/TiO_2_ NRs, (**b**) the middle portion of Al_2_O_3_/TiO_2_ NRs, (**c**) the top portion, (**d**) SAED of Al_2_O_3_/TiO_2_ NRs annealed at 500 °C, (**e**) Al_2_O_3_/TiO_2_ NRs, (**f**) the middle portion of Al_2_O_3_/TiO_2_ NRs, and (**g**) the top portion, (**h**) SAED of Al_2_O_3_/TiO_2_ NRs.

A 2D thin film of TiO_2_ NRs was generated through the reaction of TTIP with water [Reaction (1)]. Subsequently, this layer was subjected to etching with HCl. In this process, the Cl^−^ ions in HCl molecules reacted with Ti atoms to form TiCl_3_ [Reaction (2)]. Previous research [18] has indicated that TiO_2_ NRs with a preferred (002) orientation can be obtained using 6 M HCl [Figure 3a]. A cross-sectional FE-SEM image of the deposited Al_2_O_3_ passivation layer is displayed in Figure 3b. The thickness of this layer was approximately 5 nm, and this thickness value was confirmed through HR-TEM [Figure 4c]. Figure 3c shows the EDS elemental analysis results for the ALD-Al_2_O_3_ film. Mapping analysis results show that the Al_2_O_3_ film grows uniformly, and the element ratio of Al to O is 40% to 60%. The ALD process involves chemical grafting to grow an Al_2_O_3_ thin film (Figure 2). A single ALD cycle involves the following steps: First, water vapor was used to attach the OH^−^ functional groups to the surface of the TiO_2_ NRs. Subsequently, TMA gas was introduced to grow the Al_2_O_3_ thin film, after which CH_4_ was removed using Ar gas. In this study, 50 ALD cycles were conducted to grow an Al_2_O_3_ passivation layer to ensure that this layer was uniformly coated on the TiO_2_ NRs, thereby enhancing its passivation effect on the surface defects of these NRs.

Ti{OCH(CH_3_)_2_}_4_ + 2H_2_O → TiO_2_ + 4(CH_3_)_2_CHOH (1)

2Ti + 6HCl → 2TiCl_3_ + 3H_2_↑ (2)

To explore the effect of the annealing treatment on the microstructure of the Al_2_O_3_/TiO_2_ NRs, HR-TEM was conducted. Figure 4a,d display the cross-sectional HR-TEM image and SAED pattern of the Al_2_O_3_/TiO_2_ NRs produced without annealing treatment. This image reveals that the lattice spacing of the TiO_2_ (110) plane was 3.31 Å [Figure 4b]. Moreover, the lattice spacings of the Al_2_O_3_ (003) and TiO_2_ (101) planes were 4.91 Å and 3.47 Å, respectively [Figure 4c]. The analysis results revealed that in the unannealed Al_2_O_3_/TiO_2_ NR structure, the Al_2_O_3_ thin film was predominantly concentrated on the outer edges of the TiO_2_ NRs, and the Al_2_O_3_ lattice did not form [Figure 4b]. Figure 4e,h display a cross-sectional HR-TEM image and SAED of the Al_2_O_3_/TiO_2_ NR structure after this structure was annealed at 500 °C. The analysis results revealed that after annealing at 500 °C, the lattice spacing of the TiO_2_ (110) plane was 3.24 Å [Figure 4f]. In the middle section of the Al_2_O_3_/NR structure [Figure 4g], the lattice spacings of the TiO_2_ (110), Al_2_O_3_ (003), Al–Ti–O (111), and Al–Ti–O (311) planes were 3.26, 4.86, 3.89, and 2.08, respectively. These results indicate that after annealing at 500 °C, Al_2_O_3_ diffused into the TiO_2_ NRs. As shown in Figure 4g, the thickness of the interface between TiO_2_ and Al_2_O_3_ increased to 10 nm after annealing at 500 °C, which confirmed that Al, Ti, and O atoms diffused across the interface between TiO_2_ and Al_2_O_3_ to form Al–Ti–O bonds. This diffusion process resulted in the effective filling of the oxygen vacancies in TiO_2_. Thus, annealing can increase the thickness of the Al_2_O_3_/TiO_2_ interface, which reduces the oxygen vacancy defects in the TiO_2_ NRs, thereby enhancing the photodetector performance.

**Figure 5:**
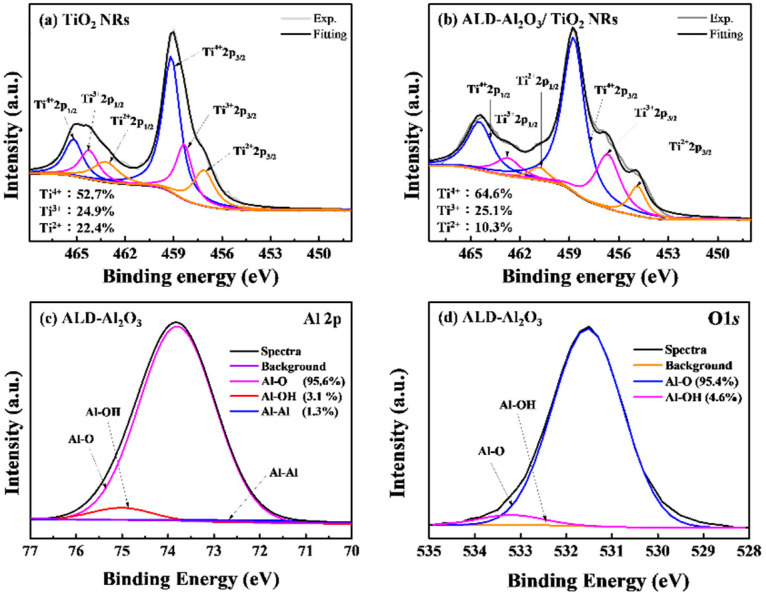
Ti *2p* spectra of the (**a**) TiO_2_ NRs, (**b**) Al_2_O_3_/TiO_2_ NRs annealed at 500 °C and (**c**) Al *2p*, (**d**) O *1s* of ALD-Al_2_O_3_ film.

Figure 5a,b show the Ti *2p* XPS spectra of the TiO_2_ NRs before and after they were passivated by the Al_2_O_3_ film. The background noise in these spectra was eliminated using the Shirley method in the Spectral Data Processor v4.5 software program, and the spectra were deconvoluted. The binding energy (*E_b_*) was calculated as follows:

*E*_*b*_ = *hv* − *E*_*z*_ − *w* (3)

where *E_z_* is the kinetic energy of the emitted electron, *w* is the work function, which represents the energy required to remove an electron from the surface of a solid, and *hv* is the incident photon energy. The binding energy calculations enabled the determination of the proportions of different oxidation states of Ti in TiO_2_, namely Ti^4+^, Ti^3+^, and Ti^2+^. Ti^3+^ and Ti^2+^ represent oxygen vacancy states, and Ti^4+^ denotes a stable state. The Ti^4+^_2p3/2_ peak was located at 459 ± 0.2 eV. As depicted in Figure 5a,b, after the TiO_2_ NRs were coated with the Al_2_O_3_ passivation film, the area proportion of the Ti^4+^ peak in the Ti *2p* XPS spectrum increased from 52.7% to 64.6%, whereas the combined area proportion of the Ti^3+^ and Ti^2+^ peaks decreased from 47.3% to 35.4%. These results indicated that the oxygen vacancy defects in the TiO_2_ NRs were reduced after they were coated with the Al_2_O_3_ layer. This improvement was attributed to the chemical passivation effect of the Al_2_O_3_ film, which reduced the surface defect density of the TiO_2_ NRs and the number of dangling bonds on their surfaces, thereby enhancing their intrinsic properties. Figure 5c,d show the Al *2p* and O *1s* XPS spectra of the Al_2_O_3_ layer. From Figure 5c, it can be observed that the area percentages of Al-O, Al-OH, and Al-Al were 95.6%, 3.1%, and 1.3%, respectively. This result shows that the bond between Al and oxygen is complete, and the Al_2_O_3_ film has few defects. However, Figure 5d again proves that the bond between oxygen and aluminum is complete, and the Al-O and Al-OH areas are 95.4% and 4.6%, respectively. In this way, a passivation layer with fewer defects can further improve the characteristics of TiO_2_ NRs UV photodetectors.

**Figure 6:**
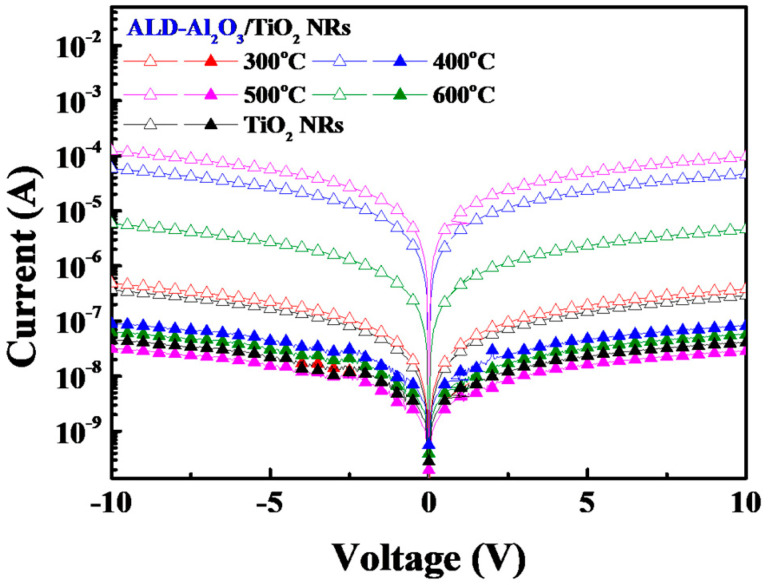
Photocurrent and dark current characteristics of SLHJ UV photodetectors with Al_2_O_3_/TiO_2_ NR structures subjected to annealing under different temperatures.

Figure 6 displays the *I*–*V* curves measured under UV illumination and dark conditions for SLHJ UV photodetectors with Al_2_O_3_/TiO_2_ NR structures subjected to annealing at different temperatures. The measurements were taken under both ultraviolet illumination and dark conditions. The results revealed that the photodetector containing an Al_2_O_3_/TiO_2_ NR structure annealed at 500 °C exhibited a photocurrent and dark current of 1.22 × 10^−5^ and 8.96 × 10^−9^ A, respectively. These results confirm that high photodetection performance can be achieved when the photodetector contains an Al_2_O_3_ passivation layer that has been deposited through ALD and annealed at an optimal temperature. To evaluate the effect of an Al_2_O_3_ passivation layer on photocurrent characteristics, the sensitivity, light-dependent resistance (LDR), and detectivity of photodetectors with and without an Al_2_O_3_ thin-film coating were compared in Table 2. The formulas for calculating these parameters are as follows:

(4) $$Sensitivity=\frac{I_{photo}}{I_{dark}}$$

(5) $$LDR=20\;\log\;(\frac{I_{photo}}{I_{dark}})$$

(6) $$Detectivity=\frac{R}{\sqrt{2\;q\;I_{dark}}}$$

where *I_photo_* is the photocurrent, *I_dark_* is the dark current, *q* is the elementary charge of an electron, and *R* is the responsivity. The calculation results indicated that the sensitivity, LDR, and detectivity of the photodetector with an Al_2_O_3_/TiO_2_ NR structure exhibited a substantial improvement in performance than those of a photodetector with TiO_2_ NRs only, with sensitivity increasing from 8 to 4380, LDR increasing from 17.7 dB to 72.8 dB, and detectivity increasing from 3.19 × 10^7^ to 1.73 × 10^10^ Jones. The chemical passivation effect of the Al_2_O_3_ film considerably enhanced the sensitivity of the TiO_2_ NRs. Therefore, the resulting UV photodetector exhibited an improved ability to detect UV light and a faster response. In general, high sensitivity correlates with high detectivity, which is a quality factor that reflects photodetectors’ ability to avoid interference from other light sources during UV detection. Moreover, the LDR is inversely proportional to the light intensity; thus, it can be used to assess the stability of photodetectors under dark conditions. A high LDR value under dark conditions indicates high device stability. Overall, the Al_2_O_3_ passivation layer improved the stability of the SLHJ UV photodetectors.

**Figure 7:**
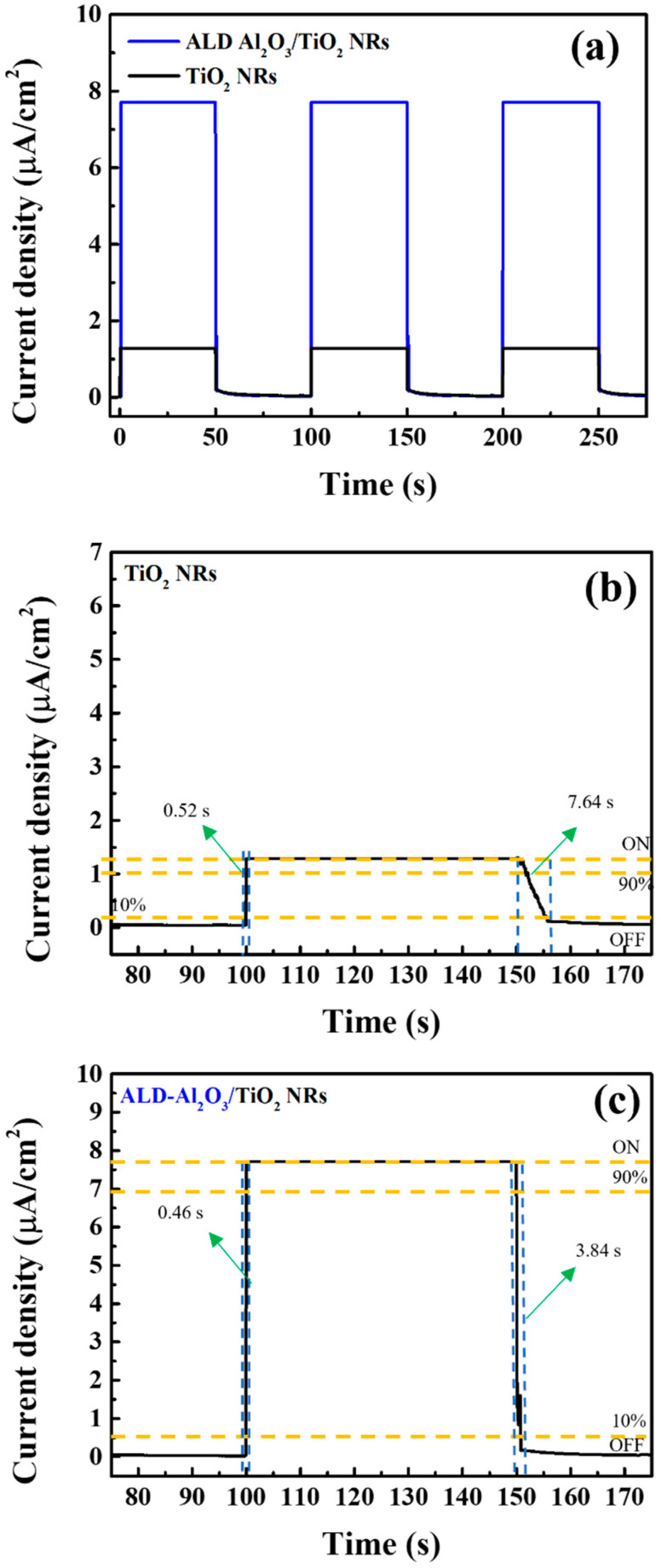
(**a**) Switching characteristics of SLHJ UV photodetectors with a TiO_2_ NR structure and Al_2_O_3_/TiO_2_ NR structure, (**b**) magnified view of the switch-on and switch-off times of the UV photodetector with the TiO_2_ NR structure, and (**c**) magnified view of the switch-on and switch-off times of the UV photodetector with the Al_2_O_3_/TiO_2_ NR structure.

Figure 7 depicts the on–off switching characteristics of UV photodetectors with and without an Al_2_O_3_ passivation layer (i.e., with an Al_2_O_3_/TiO_2_ NR structure and a TiO_2_ NR structure, respectively). During the measurement process, no external bias was applied (bias = 0), and the switching time and total measurement duration were set to 50 and 275 s, respectively. As displayed in Figure 7a, the Al_2_O_3_ passivation layer caused the photocurrent density to increase from 1.7 to 7.8 μA/cm^2^. Figure 7b,c displays magnified views of the switch-on and switch-off times of the photodetectors with a TiO_2_ NR structure and Al_2_O_3_/TiO_2_ NR structures, respectively. The switch-on time was defined as the time when the current density exceeded 90%, and the switch-off time was defined as the time when the current density dropped below 10%. The switch-on times of the photodetectors with a TiO_2_ NR structure and Al_2_O_3_/TiO_2_ NR structure were 0.52 and 0.46 s, respectively. Furthermore, their switch-off times were 7.64 and 3.84 s, respectively. These results indicate that the Al_2_O_3_ thin film considerably improves the switching characteristics of the photodetector. This improvement was attributed to the chemical passivation effect of this film, which effectively reduced the number of oxygen vacancy defects in the TiO_2_ NRs, thereby enhancing the switching characteristics of the UV photodetector.

## 4. Conclusions

This study improved the photodetection performance of UV photodetectors with SLHJ- and TiO_2_-NR-containing active layers by depositing an Al_2_O_3_ passivation layer on this active layer through ALD. The results of HR-TEM results confirmed that after the Al_2_O_3_/TiO_2_ NR structure was annealed, an Al–Ti–O interface was formed between TiO_2_ and Al_2_O_3_. This interface was formed because Al diffused into the TiO_2_ NRs during thermal annealing, thereby passivating their surface defects. XPS analyses indicated that the Al_2_O_3_ passivation layer reduced the Ti^3+^ and Ti^2+^ defects in the TiO_2_ NRs, with the combined area proportion of these two defects in the Ti *2p* XPS spectrum decreasing from 47.3% to 35.4%. This reduction in oxygen vacancy defects was attributed to the filling effect provided by the ALD-Al_2_O_3_ process. Accordingly, the sensitivity of the ALD-Al_2_O_3_/TiO_2_ NR SLHJ ultraviolet photodetector increased from 8 to 4380, and its detectivity improved from 3.19 × 10^7^ to 1.73 × 10^10^ Jones. The photocurrent-to-dark-current ratio exhibited a difference of four orders of magnitude. Additionally, the turn-on and turn-off times of the photodetector decreased by 0.06 and 3.8 s, respectively. The aforementioned results indicate that the ALD-Al_2_O_3_ passivation layer is highly effective and has the potential for future applications in enhancing the performance of photodetector devices.

## Figures and Tables

**Table 1:** The performance comparison of UV photodetectors studies.

Active Layer	λ_cut_ (nm)	Sensitivity	Detectivity (Jones)	Ref.
**Al_2_O_3_/TiO_2_ NR**	365	4380	1.73 × 10^10^	this work
**TiO_2_ NRs/Au/PTTh**	365	-	1.67 × 10^10^	[19]
**AlN/TiO_2_ nanorod**	350	1360	2.87 × 10^9^	[20]
**ZnO/TiO_2_**	365	388	1.10 × 10^10^	[21]
**AgNW/NiO TiO_2_/FTO**	365	-	1.60 × 10^10^	[22]

**Table 2:** Photocurrent, dark current, sensitivity, light-dependent resistance (LDR), and detectivity of ultraviolet (UV) photodetectors with solid–liquid heterojunction (SLHJ) and Al_2_O_3_/TiO_2_ nanorod (NR) structures annealed at various temperatures.

Annealing Temperature	I_photo_ (A)	I_dark_ (A)	Sensitivity	LDR (dB)	Detectivity (Jones)
**TiO_2_ NRs**	3.66 × 10^−7^	4.38 × 10^−8^	8	17.7	3.19 × 10^7^
**300 °C**	4.81 × 10^−7^	4.65 × 10^−8^	10	20.3	4.49 × 10^7^
**400 °C**	6.12 × 10^−5^	8.96 × 10^−8^	683	56.7	4.55 × 10^9^
**500 °C**	1.37 × 10^−4^	3.13 × 10^−8^	4380	72.8	1.73 × 10^10^
**600 °C**	5.81 × 10^−6^	7.19 × 10^−8^	81	38.1	4.77 × 10^8^

## Data Availability

The raw and processed data supporting the findings are available upon reasonable request.

## References

[1] Chatterjee A., Agnihotri V., Porwal S., Khan S., Baraik K., Ganguli T., Bose A., Raghavendra S., Dixit V.K., Sharma T.K. (2024). Impact of oxygen plasma power on the performance of Ga_2_O_3_ passivated GaN ultraviolet photodetectors. Appl. Surf. Sci..

[2] Mohammad F.K., Ramizy A., Ahmed N.M., Yam F.K., Hassan Z., Beh K.P. (2024). Fabrication and photoresponsive characteristics of ultraviolet GaN p-i-n photodetector based AlN:Al_2_O_3_ passive layer. Opt. Mater..

[3] Razeen A.S., Kotekar-Patil D., Tang X.S., Yuan G., Ong J., Radhakrishnan K., Tripathy S. (2024). Enhanced near-UV responsivity of AlGaN/GaN HEMT based photodetectors by nanohole etching of barrier surface. Mat. Sci. Semicon. Proc..

[4] Rajamanickam S., Mohammad S.M., Razak I.A., Muhammad A., Abed S.M. (2023). Enhanced sensitivity from Ag micro-flakes encapsulated Ag-doped ZnO nanorods-based UV photodetector. Mater. Res. Bull..

[5] Mohammed A.A.A., Lim W.F. (2024). High photosensitivity performance vertical structured metal-semiconductor based ultraviolet photodetector using Ga_2_O_3_ thin film sputtered on *n*-type Si(100). Mater. Sci. Eng. B.

[6] Huang J.J., Lin C.H., Ho Y.R., Chang Y.U. (2020). Aluminium oxide passivation films by liquid phase deposition for TiO_2_ ultraviolet solid–liquid heterojunction photodetectors. Surf. Coat. Technol..

[7] Zhou J.W., Qiao Q., Tan Y.F., Wu C., Hu J.W., Qiu X.F., Wu S.H., Zheng J., Wang R., Zhang C.X. (2023). The improvement of polymer photodetector based on 1D-ZnO nanorod arrays/0D-ZnO quantum dots composite film. Opt. Mater..

[8] Karagoz E., Altaf C.T., Yaman E., Yildirim I.D., Erdem E., Celebi C., Fidan M., Sankir M., Sankir N.D. (2023). Flexible metal/semiconductor/metal type photodetectors based on manganese doped ZnO nanorods. J. Alloys Compd..

[9] Abdulrahman A.F., Abd-Alghafour N.M., Almessiere M.A. (2023). A high responsivity, fast response time of ZnO nanorods UV photodetector with annealing time process. Opt. Mater..

[10] Nithya G., Kumar K.N., Shaik H., Reddy S., Sen P., Prakash N.G., Ansari M.A. (2024). Simulation and Deposition of Tungsten Oxide (WO_3_) films using DC sputtering Towards UV Photodetector for High Responsivity. Physica B.

[11] Kumar M., Dhar J.C. (2024). Enhanced UV photodetector performance using sputtered Mg-doped ZnO thin film. Opt. Mater..

[12] Chen Y.C., Chen D.B., Zeng G., Li X.X., Li Y.C., Zhao X.F., Chen N., Wang T.Y., Yang Y.G., Zhang D.W. (2023). High performance solar-blind photodetectors based on plasma-enhanced atomic layer deposition of thin Ga_2_O_3_ films annealed under different atmosphere. J. Alloys Compd..

[13] Chu S.Y., Yeh T.H., Lee C.T., Lee H.Y. (2022). Mg-doped beta-Ga_2_O_3_ films deposited by plasma-enhanced atomic layer deposition system for metal-semiconductor-metal ultraviolet C photodetectors. Mat. Sci. Semicon. Proc..

[14] Jehad A.K., Fidan M., Ünverdi Ö., Çelebi C. (2023). CVD graphene/SiC UV photodetector with enhanced spectral responsivity and response speed. Sens. Actuators A Phys..

[15] Almaviva S., Marinelli M., Milani E., Prestopino G., Tucciarone A., Verona C., Verona-Rinati G., Angelone M.L., Pillon M. (2009). Extreme UV photodetectors based on CVD single crystal diamond in a p-type/intrinsic/metal configuration. Diam. Relat. Mater..

[16] Samriti, Prateek, Joshi M.C., Gupta R.K., Prakash J. (2022). Hydrothermal synthesis and Ta doping of TiO_2_ nanorods: Effect of soaking time and doping on optical and charge transfer properties for enhanced SERS activity. Mater. Chem. Phys..

[17] Naceur J.B., Jrad F., Souiwa K., Rhouma F.B., Chtourou R. (2021). Hydrothermal reaction time effect in wettability and photoelectrochemical properties of TiO_2_ nanorods arrays films. Optik.

[18] Huang J.J., Ho Y.R., Chang Y.H., Lin C.H., Ou S.L. (2021). Al_2_O_3_-passivated TiO_2_ nanorods for solid-liquid heterojunction ultraviolet photodetectors. J. Mater. Sci..

[19] Zhang H.J., Abdiryim T., Jamal R., Liu X., Niyaz M., Xie S.Y., Liu H.L., Kadir A., Serkjan N. (2022). Coal-based carbon quantum dots-sensitized TiO_2_ NRs/PTTh heterostructure for self-powered UV detection. Appl. Surf. Sci..

[20] Huang J.J., Ho Y.R. (2023). Performance improvement of TiO_2_ nanorods ultraviolet photodetector by AlN thin film passivation. Mat. Sci. Semicon. Proc..

[21] Zhou M., Wu B., Zhang X., Cao S., Ma P., Wang K., Fan Z., Su M. (2020). Preparation and UV photoelectric properties of aligned ZnO-TiO_2_ and TiO_2_-ZnO core-shell structured heterojunction nanotubes. ACS Appl. Mater. Interfaces.

[22] Nguyen T.T., Patel M., Kim S., Mir R.A., Yi J., Dao V., Kim J. (2021). Transparent photovoltaic cells and self-powered photodetectors by TiO_2_/NiO heterojunction. J. Power Sources.

